# Targeting microglia-mediated neuroinflammation via pH-responsive lipid nanoparticle delivery of HDAC inhibitor RGFP966 in ischemic stroke

**DOI:** 10.1080/14686996.2026.2699624

**Published:** 2026-07-16

**Authors:** Yaqi Liu, Biao Luo, Yun Xu, Dexiang Liu, Qiujing Wang

**Affiliations:** a Department of Cerebrovascular Surgery, The Third Affiliated Hospital of Sun Yat-Sen University, Guangzhou, China; b Department of Interventional Operating Room, The Third Affiliated Hospital of Sun Yat-Sen University, Guangzhou, China

**Keywords:** Ischemic stroke, ph-responsive LNP, microglia, neuroinflammation, HDAC

## Abstract

Ischemic stroke (IS) remains a leading cause of global disability and mortality, with limited effective anti-inflammatory interventions. Targeting histone deacetylase 3 (HDAC3) to reprogram microglial polarization holds great therapeutic promise, but the HDAC3-specific inhibitor RGFP966 suffers from poor blood-brain barrier (BBB) penetration and systemic toxicity. Herein, we developed a pH-responsive brain-targeted lipid nanoparticle (CD-TLNP@RGFP966) by integrating T7 peptide modification for BBB transcytosis and cinnamaldehyde-α-cyclodextrin (CA-αCD) encapsulation for acidic lesion-triggered drug release. CD-TLNP@RGFP966 exhibited favorable colloidal stability, biosafety, and pH-dependent release behavior. It efficiently crossed the BBB, accumulated in ischemic brain tissue, promoted microglial M2 polarization, attenuated neuroinflammation, reduced infarct volume, and improved neurological function in tMCAO mice. This study provides a novel targeted nanocarrier strategy for the anti-inflammatory treatment of IS.

## IMPACT STATEMENT

We constructed pH-responsive brain-targeted CD-TLNP@RGFP966 using T7-peptide and acetal-based CA-αCD, enabling lesion-specific delivery, promoting microglial M2 polarization and neuroinflammation relief for improved ischemic stroke recovery.

## GRAPHICAL ABSTRACT

**Figure:**
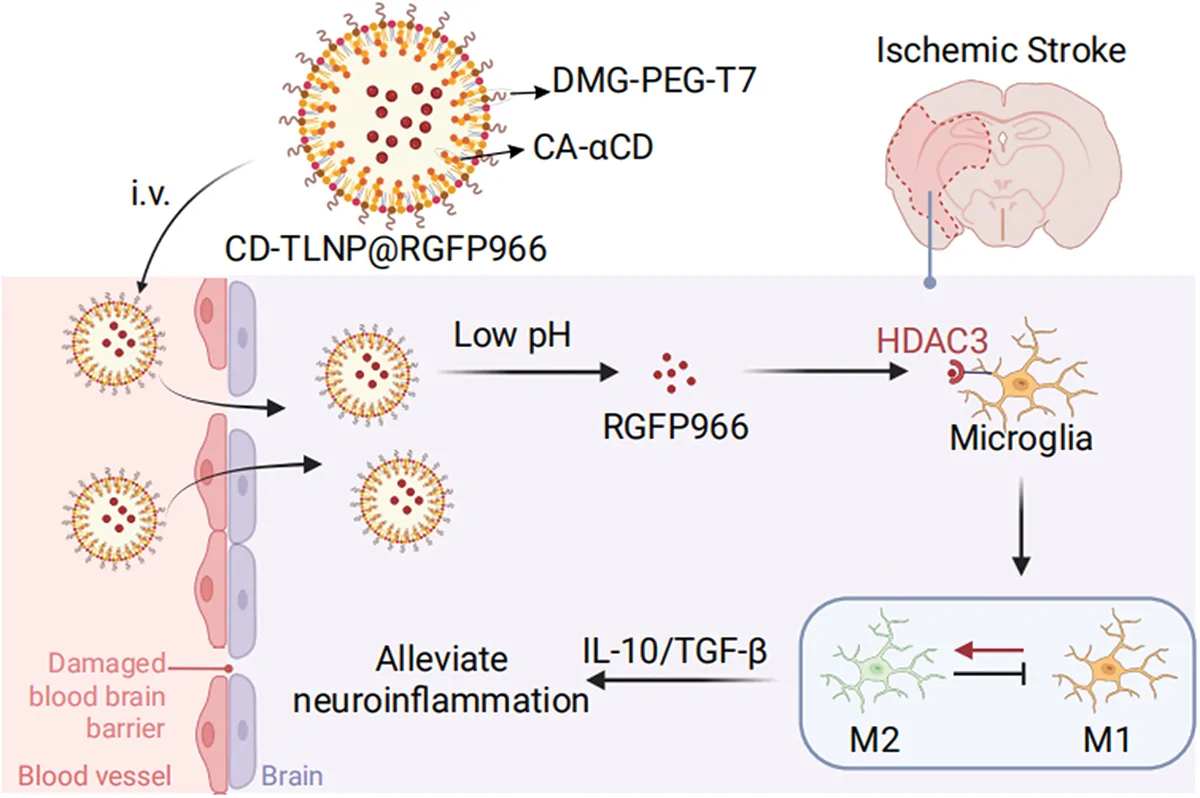

## 1. Introduction

Ischemic stroke (IS) is a critical central nervous system disease with high rates of disability, mortality, and recurrence worldwide [1]. Its pathological process is complex, involving blood-brain barrier (BBB) disruption, oxidative stress, neuroinflammatory cascade activation, and neuronal apoptosis, which are closely interrelated and jointly contribute to brain tissue damage [2–4]. While early revascularization through thrombolysis or thrombectomy constitutes the current standard of care for IS, its clinical utility is constrained by a narrow treatment window, the inherent hazard of reperfusion injury, and the lack of effective anti-inflammatory interventions-all of which collectively contribute to suboptimal patient recovery [5–7]. Consequently, there is an urgent need for therapeutic strategies that can precisely intervene in the neuroinflammatory cascade to improve prognosis.

Microglia serve as the resident innate immune cells of the central nervous system, and their phenotypic polarization profile largely dictates the progression of neuroinflammation. Following cerebral ischemia, microglia become rapidly activated and predominantly polarize toward the pro-inflammatory M1 phenotype [8–10]. Activated M1 microglia robustly secrete pro-inflammatory mediators including TNF-α and IL-1β, which amplify inflammatory cascades and exacerbate neuronal injury. Shifting microglia toward an anti-inflammatory M2 phenotype can suppress inflammation and promote repair. Histone deacetylase 3 (HDAC3) acts as a critical modulator governing microglial activation and phenotypic switching [11–13]. Ischemic insult triggers abnormal upregulation of HDAC3 in microglia, which further drives exaggerated microglial activation and pro-inflammatory phenotypic transition [14]. In this context, targeted inhibition of HDAC3 is capable of reprogramming microglial immune phenotypes and halting neuroinflammatory progression, rendering HDAC3 a promising therapeutic target for IS. However, the HDAC3-specific inhibitor RGFP966 suffers from poor solubility, limited brain penetration, and systemic toxicity, hindering its clinical application [15–17].

The ischemic penumbra, a key therapeutic target in IS,exhibits a characteristic acidic microenvironment, which provides a natural rationale for designing pH-responsive drug carriers [18–20]. This acidic condition is capable of inducing structural alterations in a range of pH-sensitive functional groups, including amino, carboxyl, imidazole and acetal groups, which further enables intelligent carrier disassembly and site-specific controlled drug release at the lesion site [21–23]. Notably, α-Cyclodextrin (αCD) offers excellent biocompatibility and safety, making it an attractive molecular scaffold for constructing brain-targeted nanocarriers [24–26]. Leveraging these properties, we designed and synthesized a pH-responsive derivative, cinnamaldehyde-α-cyclodextrin (CA-αCD). In this structure, the acetal linkage was introduced as a pH-sensitive functional moiety grafted onto the α-cyclodextrin backbone.

Meanwhile, T7 peptide (HAIYPRH), a well-characterized BBB-penetrating peptide, specifically binds to the transferrin receptor (TfR) highly expressed on the surface of brain microvascular endothelial cells-a key component of the BBB [27]. This specific binding enables the T7 peptide to mediate receptor-mediated transcytosis across the BBB, effectively facilitating the nanoparticles penetration into the brain parenchyma and improving their targeted accumulation at ischemic lesions [28–30]. Thus, In this study, we constructed T7 peptide-modified lipid nanoparticles and encapsulated the CA-αCD via hydrophilic-hydrophobic interactions, thereby assembling a brain-targeted, pH-triggered delivery system. This system was further loaded with RGFP966 to form CD-TLNP@RGFP966, aiming to alleviate post-stroke neuroinflammation. The results demonstrated that CD-TLNP@RGFP966 exhibited favorable colloidal stability, good biosafety, and pH-responsive drug release. It efficiently crossed the BBB, accumulated in the ischemic hemisphere, promoted microglial polarization toward the M2 phenotype, attenuated neuroinflammation, and ultimately improved functional recovery after stroke. This study aims to develop an efficient and safe pH-responsive brain-targeted nano-delivery system, providing a novel carrier strategy and experimental basis for the targeted anti-inflammatory treatment of IS.

## 2. Materials and methods

### 2.1. Materials

Cinnamaldehyde (CA), sodium bicarbonate (NaHCO_3_), α-cyclodextrin (α‑CD), trimethyl orthoformate, pyridinium p‑toluenesulfonate (PPTS), ethyl acetate, magnesium sulfate (MgSO_4_), acetone, dimethyl sulfoxide (DMSO), methanol, absolute ethanol, sodium citrate buffer (0.1 M, pH 4.0), and the fluorescent dye DiR were obtained from Aladdin (China). 1,2‑distearoyl‑sn‑glycero‑3‑phosphocholine (DSPC) and DLin‑MC3‑DMA were purchased from AVT (China). DMG‑PEG2000‑T7 was obtained from SunLipo NanoTech (China). RGFP966 and 2,3,5‑triphenyltetrazolium chloride (TTC) were sourced from MedChemExpress (MCE, U.S.A.). Cell Counting Kit‑8 (CCK‑8), BCA protein assay kit, bovine serum albumin (BSA), and phosphate‑buffered saline (PBS) were purchased from Beyotime Biotechnology (China). Dulbecco’s Modified Eagle Medium (DMEM) and fetal bovine serum (FBS) were obtained from Gibco (U.S.A.). The following antibodies were used: anti‑CD16/CD32 (AB171202), Alexa Fluor® 647‑conjugated goat anti‑rat IgG (AB150157), Alexa Fluor® 488‑conjugated goat anti‑mouse IgG (AB150113), anti‑Iba1 (AB178846), Alexa Fluor® 647‑conjugated goat anti‑rabbit IgG (AB150077) (all from Abcam, UK); anti‑CD86 (105030) and anti‑CD206 (162505) (both from BioLegend, U.S.A.); anti‑HDAC3 (AB32369), anti‑Bcl‑2 (AB182858), anti‑Bax (AB32503), and anti‑β‑actin (AB8227) (all from Abcam, UK). BV2 and bEnd.3 were purchased from the American Type Culture Collection (ATCC). C57BL/6J mice (male, 22–25 g) were purchased from the Experimental Animal Center of Sun Yat-sen University. All animal experiments complied with the ARRIVE guidelines and were approved by the Experimental Animal Management and Use Committee and Experimental Animal Ethics Committee of Sun Yat-Sun University and complied with international animal health guidelines.

### 2.2. Synthesis of CA-αCD

The CA-αCD was prepared according to the method reported in the literature [31]. Briefly, cinnamal acetal (CAA) was synthesized by refluxing cinnamaldehyde (1.0 g), trimethyl orthoformate (3.9 g), and PPTS (0.5 g) in methanol (50 mL) at 62°C for 3 h. After quenching the reaction with saturated NaHCO_3_ solution, the mixture was extracted with ethyl acetate, and the organic phase was collected and dried over MgSO_4_. Upon removal of the solvent under reduced pressure, a yellow liquid product (CAA) was obtained. Subsequently, α-cyclodextrin (1.0 g) and CAA (2.2 g) were dissolved in anhydrous DMSO (10 mL) with PPTS (0.20 g) and stirred at 60°C under Ar for 7 h. The product was precipitated in cold acetone, collected, and dried under vacuum to give CA-αCD.

### 2.3. Preparation of CD-TLNP@RGFP966

T7 peptide-modified lipid nanoparticles (TLNP) were prepared by rapidly mixing an ethanol phase containing DLin-MC3-DMA, DSPC, cholesterol, and DMG-PEG2000-T7 (molar ratio 20:40:38.5:1.5) with a 0.1 M sodium citrate buffer (pH 4.0) at a 1:3 (v/v) ratio. The resulting mixture was dialyzed against PBS (pH 7.4) for 24 hours, and the final TLNP were collected. To prepare the pH-responsive variant (CD-TLNP), 5 µg of CA-αCD was added to the ethanol phase prior to mixing, with all other steps identical to the TLNP preparation. For the drug-loaded formulation (CD-TLNP@RGFP966), 1 µM RGFP966 was additionally incorporated into the ethanol phase alongside CA-αCD, following the same procedure. For the preparation of DiR-labeled CD-TLNP@RGFP966, 1 µg of the DiR fluorescent dye was added to the ethanol phase prior to mixing.

The mean diameter and surface potential of nanoparticles were characterized using Zetasizer Nano ZS (Malvern, UK). The morphology of CD-TLNP@RGFP966 was observed by TEM (JEOL, Japan). The encapsulation efficiency (EE) of RGFP966 and its in vitro release profile were analyzed by UV-Vis spectrophotometry at 330 nm. For the drug release study, CD-TLNP@RGFP966 was diluted in PBS buffers (pH 7.4 and 6.5) and incubated at 37°C with shaking at 100 rpm. At designated time intervals, samples were withdrawn and treated with Triton X-100 to disrupt the nanoparticles. After centrifugation, the supernatant was assayed to determine the released RGFP966 concentration, and the cumulative release percentage was calculated.

### 2.4. In vitro biocompatibility and BBB permeability of CD-TLNP@RGFP966

Cytotoxicity was determined via a CCK-8 cell viability assay. BV2 cells were plated in 96-well plates (1 × 10^4^ cells/well) and cultured overnight. The cells were then exposed to increasing concentrations of CD-TLNP@RGFP966 for 24 hours. Post-treatment, 10 µL of CCK-8 reagent was added to each well, and the plates were incubated for 1 hours. The absorbance of the formazan product was measured at 450 nm using a microplate reader (Thermo, USA). Cell viability was expressed as a percentage relative to the untreated control group.

The ability of DiR-labeled CD-TLNP@RGFP966 to cross the BBB was assessed using an in vitro BBB model. Briefly, A co-culture model was established with bEnd.3 and BV2 cells seeded in the upper and lower chambers at 1 × 10^4^ cells per well, respectively. The nanoparticles were then added to the apical chamber. Following a 24-hour incubation, the fluorescence intensity in the lower chamber was measured to quantify the extent of transendothelial transport.

### 2.5. Effect of CD-TLNP@RGFP966 on M1/M2 polarization of microglia in vitro

BV2 cells were seeded in 96-well plates (1 × 10^4^ cells/well) and subjected to oxygen-glucose deprivation/reoxygenation (OGD/R) to simulate ischemic injury. For the OGD phase, cells were incubated in glucose-free DMEM under a hypoxic atmosphere (95% N_2_, 5% CO_2_) at 37°C for 12 hours. This was followed by the reoxygenation phase, in which the medium was replaced with complete DMEM and cultured under normoxic conditions at 37°C for another 12 hours. Subsequently, the cells were treated with CD-TLNP@RGFP966 (RGFP966 final concentration: 100 nM) for 24 hours. After treatment, cells were collected, washed with cold PBS, and stained on ice for 30 minutes with fluorescently conjugated antibodies against CD86 and CD206. Following a wash to remove unbound antibodies, the cells were resuspended in buffer and analyzed by flow cytometry (Attune, USA) to determine M1/M2 polarization status.

Following the aforementioned treatments, BV2 cells were collected and lysed to extract total protein. The protein concentrations were determined using a BCA assay. Subsequently, equal amounts of protein were separated by SDS-PAGE and transferred onto PVDF membranes. After blocking, the membranes were incubated overnight at 4°C with primary antibodies against HDAC3, Bcl-2 and Bax, followed by incubation with appropriate HRP-conjugated secondary antibodies. Protein bands were visualized using an enhanced chemiluminescence (ECL) detection system, and band intensities were quantified with ImageJ software.

### 2.6. In vivo biosafety of CD-TLNP@RGFP966

To evaluate the systemic safety of CD-TLNP@RGFP966, healthy mice were randomly grouped and intravenously administered the nanoparticles (100 µL, containing 100 nM RGFP966). Peripheral blood was collected at 0.5 and 14 days post-injection for complete blood count and serum biochemistry analysis. On day 14, mice were euthanized, and heart, liver, spleen, lungs, and kidneys were harvested, fixed, and processed for hematoxylin and eosin (H&E) staining to assess histopathological changes.

### 2.7. Brain targeting of CD-TLNP@RGFP966

The transient middle cerebral artery occlusion (tMCAO) model was established in male C57BL/6 mice as previously described, involving 60 minutes of ischemia followed by reperfusion. Briefly, male C57BL/6 mice were anesthetized and a silicon-coated nylon monofilament (diameter: 0.21 ± 0.02 mm; Doccol Corporation, U.S.A.) was inserted via the external carotid artery into the internal carotid artery to occlude the origin of the middle cerebral artery. Successful occlusion was confirmed by a ≥ 70% reduction in regional cerebral blood flow measured by laser Doppler flowmetry (RWD Life Science, China). For the biodistribution study, tMCAO mice were intravenously administered DiR-labeled CD-TLNP@RGFP966 (100 µL, containing 100 nM RGFP966) at a specified time post-reperfusion. At predetermined time points post-injection, mice were euthanized and transcardially perfused with PBS. Brain, heart, liver, spleen, lungs, and kidneys were then harvested, rinsed, and immediately subjected to ex vivo fluorescence imaging using an IVIS spectrum system (PerkinElmer, USA) to quantify the DiR fluorescence signal in each organ.

**Scheme 1.**
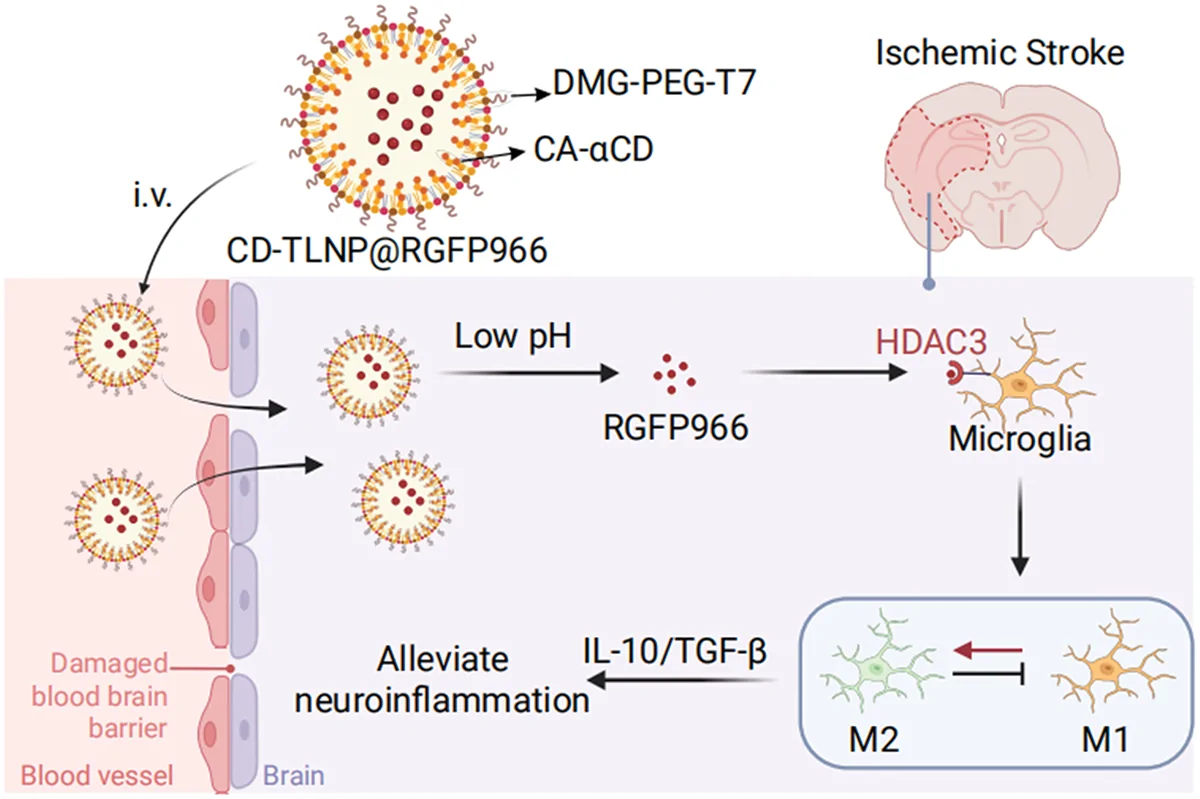
Schematic illustration of the fabrication of CD-TLNP@RGFP966 and its therapeutic mechanism in alleviating neuroinflammation following ischemic stroke.

### 2.8. Therapeutic effects of CD-TLNP@RGFP966 against IS

1 hour after tMCAO surgery, mice that met the inclusion criteria (based on neurological scores) were randomly assigned to groups. They received a single intravenous injection of CD-TLNP@RGFP966 (100 µL, containing 100 nM RGFP966) via the tail vein. Body weight and survival rates were monitored every 48 hours. At 25 hours post-modeling, mice were euthanized. The brains were rapidly removed, rinsed in cold saline, and sliced into 2-mm thick coronal sections using a brain matrix. The sections were incubated in 2% TTC solution at 37°C in the dark for 20 minutes, then fixed in 4% paraformaldehyde. The stained sections were scanned, and the infarct volume was quantified with ImageJ software. Brain water content was determined by the wet-dry weight method.

### 2.9. Immunofluorescence

Microglial polarization was assessed by immunofluorescence staining. Briefly, paraffin-embedded brain sections (10 μm thick) were deparaffinized, rehydrated, and subjected to antigen retrieval. After blocking with 5% BSA in PBS for 1 hour at room temperature, the sections were incubated overnight at 4°C with the following primary antibodies diluted in blocking buffer: rat anti-CD206 (1:200), and rabbit anti-Iba1 (1:200). The following day, sections were washed and incubated for 1 hour at room temperature with species-appropriate secondary antibodies. After counterstaining nuclei with DAPI, the sections were mounted with an anti-fade mounting medium. Fluorescent images were captured using a fluorescence microscope or a confocal laser scanning microscope (Zeiss, Germany).

At 25 hours after model establishment, brain tissues from the ischemic hemisphere were collected and homogenized in TRIzol reagent for total RNA extraction, according to the manufacturer’s protocol. Complementary DNA (cDNA) was synthesized using a reverse transcription kit. The mRNA expression levels of TGF-β, IL-10, IL-1β and IL-6 were quantified by RT-qPCR using SYBR Green Master Mix on a StepOnePlus system. GAPDH served as the endogenous control. In parallel, primary microglia were isolated from brain using a magnetic-activated cell sorting kit with anti-CD11b microbeads. Total RNA was extracted from the purified microglia, and the expression level of HDAC3 mRNA was determined by RT-qPCR as described above.

### 2.10. Neurological functional assessment

Long-term neurological function was evaluated at 7 days after tMCAO surgery. Sensorimotor deficits were first assessed using the modified Neurological Severity Score (mNSS) and the Bederson scoring system. Cognitive function, specifically spatial learning and memory, was then evaluated in the Morris water maze (MWM) test. The escape latency to find the hidden platform during the acquisition phase and the time spent in the target quadrant during the probe trial were recorded and analyzed.

### 2.11. Statistical analysis

The statistical significance was analyzed with One-way or two-way ANOVA determined by Tukey post-hoc tests using GraphPad Prism 8.2.1. All values were expressed as the mean ± standard error of the mean (SEM). ns, no statistical significance, *p < 0.05, **p < 0.01, ***p < 0.001.

## 3. Results and discussion

### 3.1. Preparation and characterization of CD-TLNP@RGFP966

**Figure 1.**
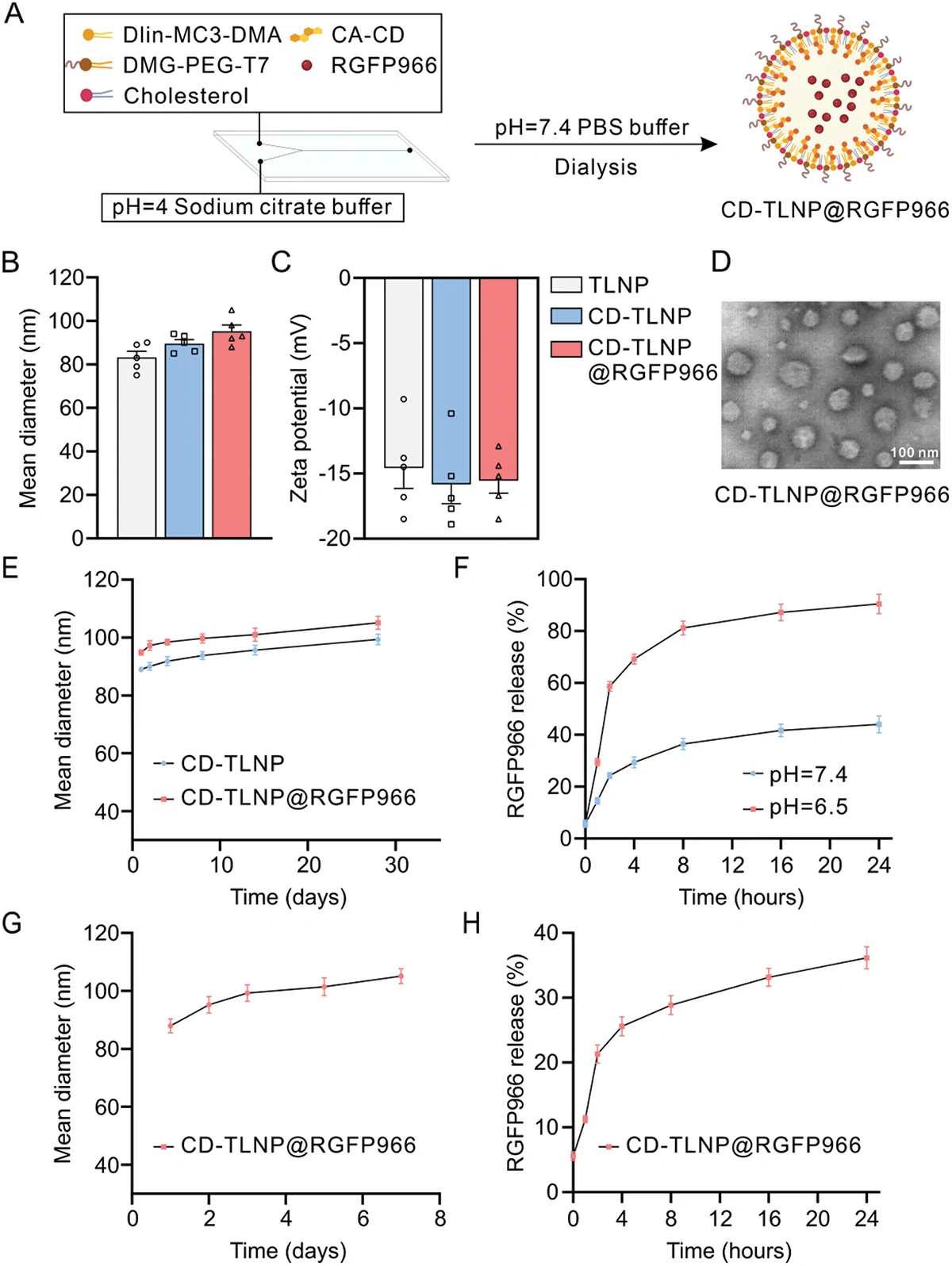
Preparation and characterization of CD-TLNP@RGFP966. (A) schematic diagram for CD-TLNP@RGFP966. (B) dynamic light scattering and (C) Zeta potential analyses of TLNP (no ROS response), CD-TLNP (ROS response) and CD-TLNP@RGFP966. (D) TEM image of CD-TLNP@RGFP966. (E) long-term stability of the nanoparticles in PBS (pH = 7.4) at 4°C. (F) in vitro release behavior of RGFP966 in different pH PBS conditions. (G) the stability of CD-TLNP@RGFP966 in PBS (10% FBS, pH = 7.4) at 4°C. (H) in vitro release behavior of RGFP966 in PBS (10% FBS, pH = 7.4). *N* = 5. Data were expressed as mean ± SEM.

We fabricated the pH-responsive, brain-targeted lipid nanoparticle delivery system CD-TLNP@RGFP966 via microfluidics (Figure 1(A)). The formulation was rationally designed by integrating pH-responsive CA-αCD and DMG-PEG-T7 into the lipid bilayer, with the aim of enabling specific response to the acidic pathological microenvironment of IS while facilitating efficient BBB penetration. Dynamic light scattering and zeta potential analyses revealed that CD-TLNP@RGFP966 had an average particle size of approximately 96 nm with a negatively charged surface (−15.5 mV), and transmission electron microscopy confirmed its regular spherical morphology (Figure 1(B–D)). The formulation showed excellent long-term physical stability, with no significant change in particle size after 28 days of storage at 4°C (Figure 1(E)). This indicates that CD-TLNP@RGFP966 remains stable when stored at 4°C, supporting its potential for clinical use. In vitro release studies showed that under physiological conditions (pH 7.4), drug release was slow, with only 44.2% released after 24 h (Figure 1(F)). In contrast, under acidic conditions (pH 6.5) that mimic the ischemic penumbra, release was rapid, reaching 81.2% within 8 h. This differential release profile is beneficial: slow release at neutral pH may reduce off-target toxicity and improve bioavailability, while rapid release in acidic environments is achieved through acid-induced hydrolysis of the acetal linkage in CA-αCD, which destabilizes the nanoparticles for localized drug delivery. The stability of CD-TLNP@RGFP966 and the release behavior of RGFP966 were further investigated in PBS containing 10% FBS. The results indicated that the average particle size did not change significantly over 7 days, demonstrating good stability (Figure 1(G)). Moreover, the release profile of RGFP966 in PBS with 10% FBS was similar to that observed in PBS buffer (Figure 1(H)). These findings suggest that the nanoparticles remain stable under physiological conditions, thereby providing a favorable basis for drug release specifically at the acidic microenvironment of the lesion site.

### 3.2. CD-TLNP@RGFP966 promoted microglial M2 polarization

**Figure 2.**
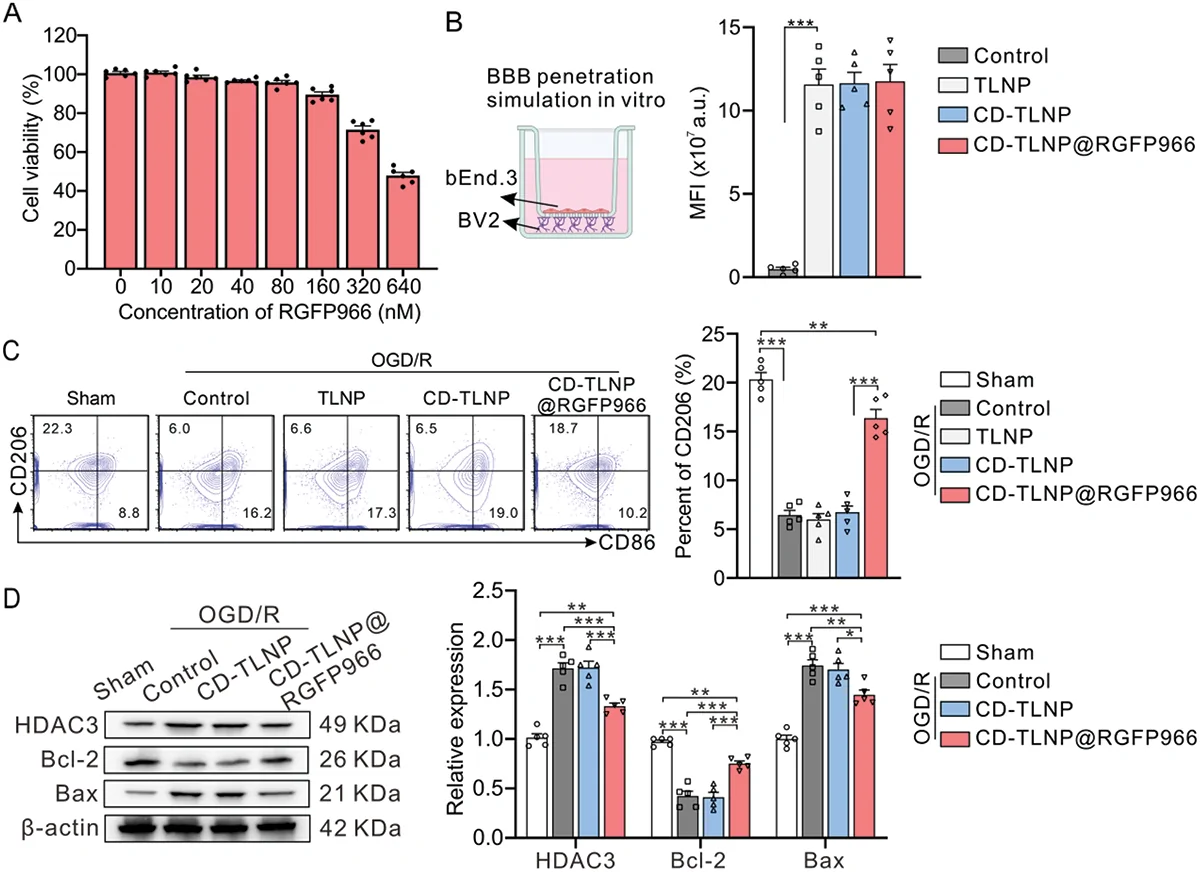
In vitro induction of microglial M2 polarization and biosafety evaluation of CD-TLNP@RGFP966. (A) the biosafety of CD-TLNP@RGFP966 was evaluated using BV2 through the CCK-8 kit. (*n* = 6). (B) fluorescence intensity of DiR-labeled-nanopartilce in the lower chamber (*n* = 5). (C) after co-incubating the OGD/R-treated BV2 with CD-TLNP@RGFP966 for 24 hours, the polarization of BV2 was detected by flow cytometry (*n* = 5). (D) the expression levels of HDAC3, Bcl-2 and Bax in BV2 were detected by Western blotting (*n* = 5). Data were expressed as mean ± SEM. *p < .05, **p < .01, ***p < .001.

First, the biosafety of CD-TLNP@RGFP966 was evaluated at the cellular level. The CCK-8 assay confirmed negligible cytotoxicity against BV2 cells at RGFP966 concentrations up to 160 nM, with a decline in cell viability observed only at 320 nM or higher (Figure 2(A)). This concentration-dependent safety profile likely reflects the intrinsic cytostatic effect of HDAC3 inhibition at supra-therapeutic levels, while the low-dose safety is attributable to the sustained release property of the nanocarrier, which prevents burst release and maintains intracellular drug concentrations within a tolerable range. Having established its biocompatibility, we evaluated its ability to cross the BBB. In a Transwell-based BBB model, T7-modified nanoparticles (TLNP, CD-TLNP, and CD-TLNP@RGFP966) showed significantly higher transport efficiency than Control, validating the T7 peptide’s role in facilitating BBB penetration (Figure 2(B)). Mechanistically, the T7 peptide specifically binds to the TfR, which is highly expressed on brain microvascular endothelial cells. This binding triggers clathrin-mediated endocytosis followed by transcytosis, allowing the nanoparticles to traverse the BBB without disrupting its integrity. The enhanced transport efficiency is therefore attributed to the receptor-mediated active transport pathway, which bypasses the paracellular route that is typically restricted by tight junctions [29,30]. Then, we examined the effect of CD-TLNP@RGFP966 on microglial polarization using BV2 cells subjected to oxygen-glucose deprivation/reoxygenation (OGD/R). Treatment with CD-TLNP@RGFP966 significantly increased the proportion of CD206^+^ (M2 marker) cells while decreasing CD86^+^ (M1 marker) expression, effectively driving microglia toward the anti-inflammatory M2 phenotype (Figure 2(C)). Mechanistically, this effect was linked to HDAC3 inhibition. Western blot analysis showed that OGD/R injury upregulated HDAC3 and the pro-apoptotic effector Bax, while downregulating Bcl-2, leading to an imbalance in the Bcl-2/Bax ratio and promoting microglial apoptosis and pro-inflammatory activation [32,33] (Figure 2(D)). The elevated HDAC3 under OGD/R conditions likely exacerbates neuronal damage by deacetylating p53 and other apoptotic regulators, shifting the balance toward Bax-dominant mitochondrial outer membrane permeabilization [14]. CD-TLNP@RGFP966 effectively normalized this profile by suppressing HDAC3 expression (reduced by 58% compared to the OGD/R group) and restoring the Bcl-2/Bax ratio (increased by 2.3-fold), suggesting that the nanocarrier inhibits microglial apoptosis while concurrently steering polarization toward the M2 phenotype. Collectively, these results indicate that CD-TLNP@RGFP966 is a safe, BBB-penetrant nanoformulation that mitigates microglial-mediated inflammation and apoptosis via targeted HDAC3 inhibition, providing a strong mechanistic rationale for in vivo stroke therapy.

### 3.3. CD-TLNP@RGFP966 exhibited excellent brain targeting capability and safety

**Figure 3.**
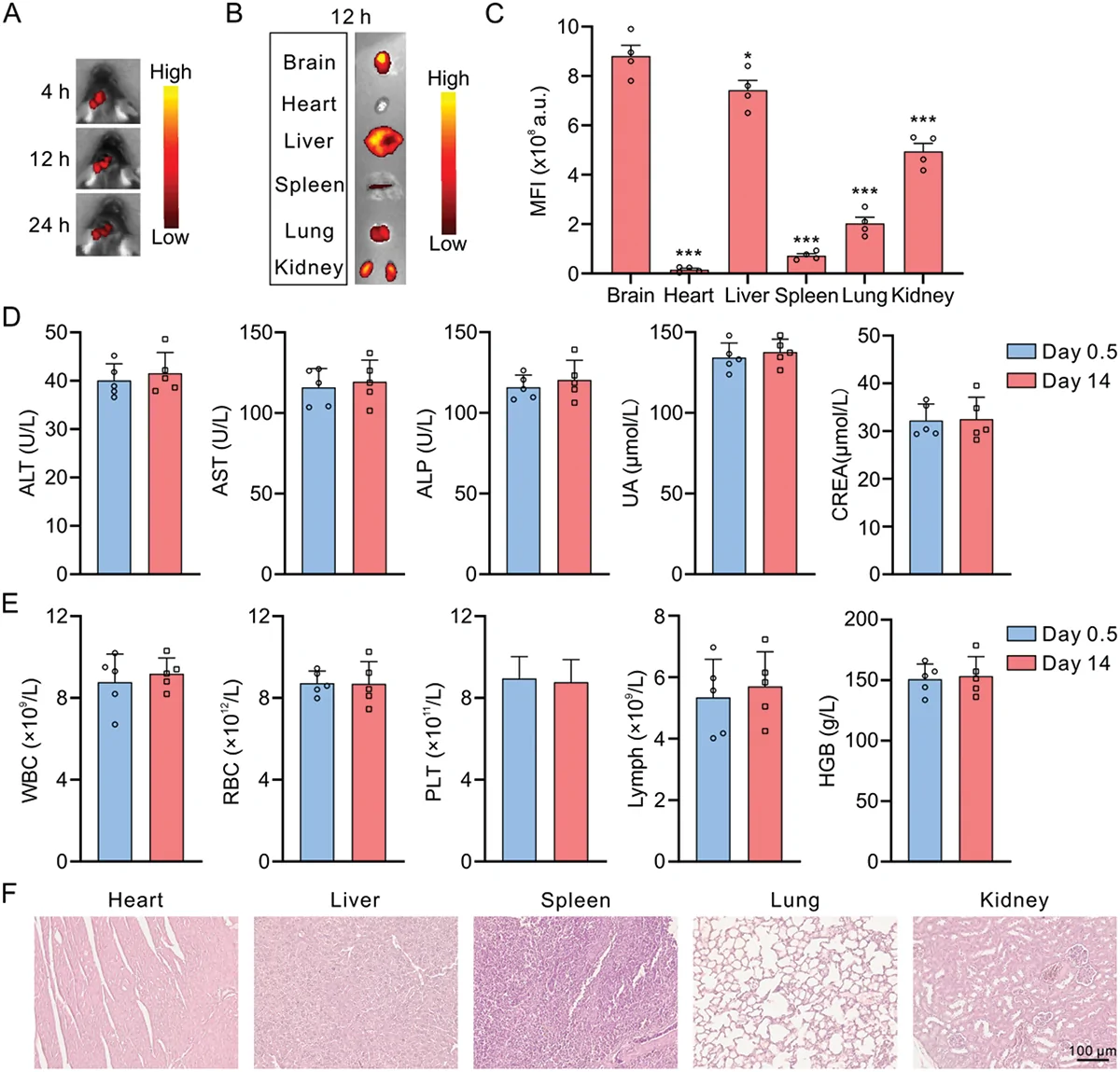
In vivo distribution and safety assessment of CD-TLNP@RGFP966. (A) Representative imaging of the brain showing the DiR-labeled-CD-TLNP@RGFP966 distribution at different time points after intravenous injection in tMCAO mice. (B) Fluorescence images of organs and (C) fluorescence intensity at 12 hours post injection (*n* = 4). (D) Blood biochemical (ALT, AST, ALP, UA, CREA) and (E) blood routine count (WBC, RBC, PLT, lymph, HBG) at 0.5 and 14 days post injection of CD-TLNP@RGFP966 (*n* = 5). (F) HE staining of the heart, liver, spleen, lung and kidney at 14 days post injection of CD-TLNP@RGFP966. Data were expressed as mean ± SEM. *p < .05, **p < .001.

The biodistribution and safety of CD-TLNP@RGFP966 were rigorously evaluated in vivo. Fluorescent imaging showed targeted brain delivery, with initial enrichment detectable at 4 hours and peak accumulation achieved at 12 hours post-injection, after which the fluorescence intensity gradually decreased but remained detectable at 24 hours, indicating that the nanocarrier can maintain a stable concentration in the brain for a prolonged period (Figure 3(A)). The observed time-course profile arises because T7 peptide-guided receptor transcytosis requires adequate time to traverse the BBB efficiently. Persistent receptor recycling and nanoparticle endocytosis together drive maximal brain infiltration at approximately 12 h. Notably, ex vivo quantification at 12 hours confirmed significantly greater nanoparticle retention in the brain compared to all major peripheral organs, underscoring its target specificity (Figure 3(B,C)). These results collectively demonstrate that the dual-functional design of CD-TLNP@RGFP966 achieves both spatial and temporal control over drug delivery, ensuring high therapeutic efficacy at the ischemic site while minimizing systemic exposure and associated toxicity.

Moreover, the formulation exhibited an excellent systemic safety profile. Keyhematological indices (WBC, RBC, PLT, Lymph, HBG) and serum biochemical indices (ALT, AST, ALP, UA, CREA) remained within normal ranges at both early (0.5 d) and late (14 d) time points, indicating that CD-TLNP@RGFP966 does not cause hematological abnormalities or liver/kidney function damage (Figure 3(D,E)). This was corroborated by HE staining, which showed no evidence of tissue damage, inflammatory cell infiltration, or abnormal morphological changes in the heart, liver, spleen, lungs, or kidneys, further confirming the good in vivo biosafety of the nanocarrier (Figure 3(F)). Taken together, CD-TLNP@RGFP966 achieves effective brain-targeted delivery without eliciting significant systemic or organ toxicity, ensuring a wide therapeutic window for further treatment studies.

### 3.4. CD-TLNP@RGFP966 alleviated cerebral damage in tMCAO mice

**Figure 4.**
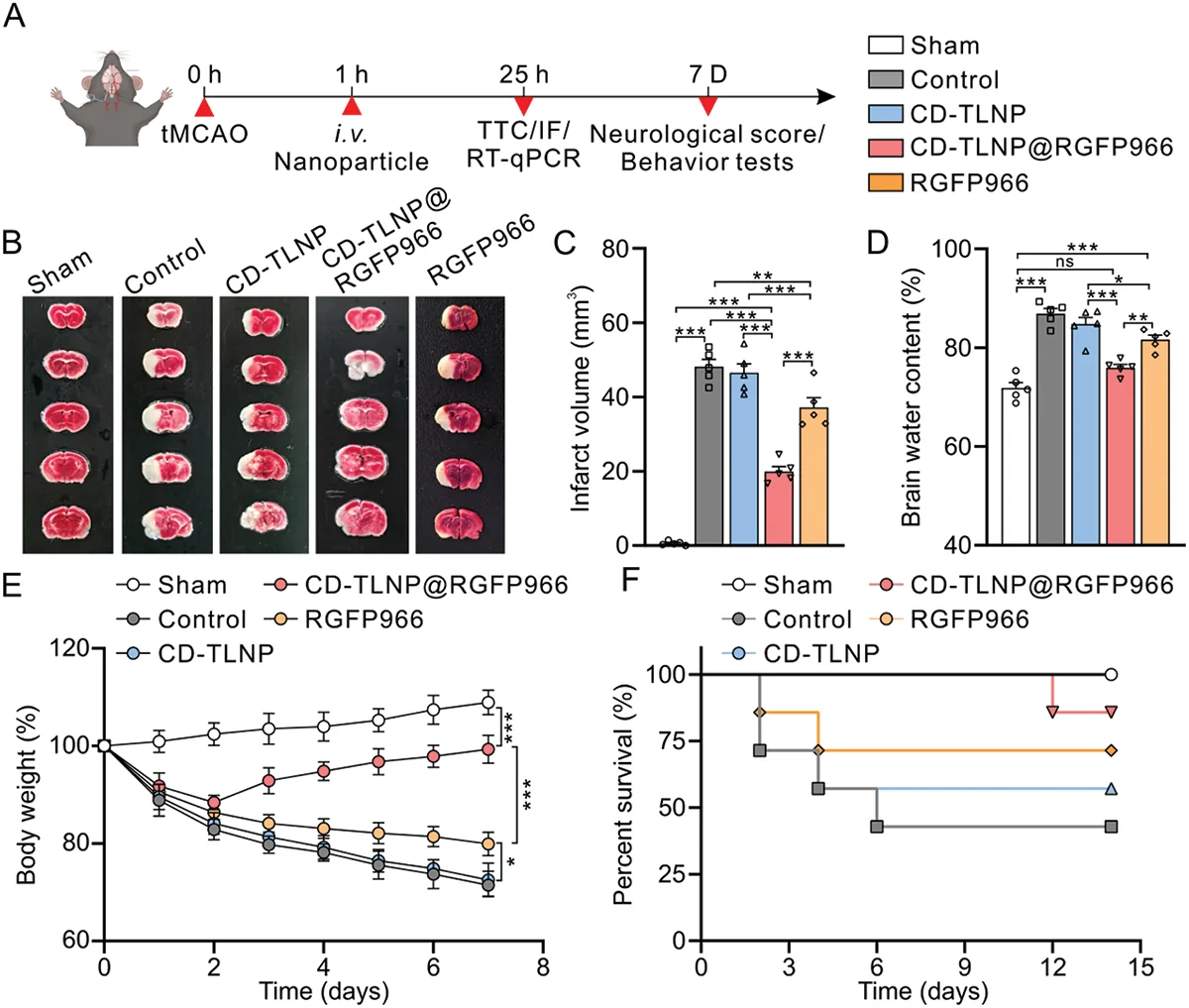
CD-TLNP@RGFP966 alleviated brain injury in tMCAO mice. (A) Experimental protocol for the tMCAO mouse model. (B) Representative images and (C) Quantitative analysis of infarct volume after different treatments based on TTC stained brain slices (*n* = 5). (D) Quantitative analysis of brain water content in different groups (*n* = 5). (E) body weight and (F) Survival time of mice in different groups (*n* = 7). Data were expressed as mean ± SEM. ns, no statistical significance, *p < .05, **p < .01, ***p < .001.

The neuroprotective efficacy of CD-TLNP@RGFP966 was decisively confirmed in the acute phaseof IS using the tMCAO mouse model (Figure 4(A)). Treatment was administered intravenously 1 hour post-tMCAO. TTC staining at 25 hours revealed a significant reduction in infarct volume in the CD-TLNP@RGFP966 group (19.8 mm^3^), which was approximately 60% and 47% smaller than that in the model Control (48.2 mm^3^) and free RGFP966 (37.2 mm^3^) groups, respectively (Figure 4(B,C)). This pronounced difference can be mechanistically attributed to two synergistic factors: (i) the T7 peptide-mediated active targeting substantially increases the local concentration of RGFP966 within the ischemic lesion compared to free drug diffusion, and (ii) the pH-responsive acetal bond ensures controlled release specifically within the acidic penumbra, avoiding premature drug loss during circulation. The superior efficacy of CD-TLNP@RGFP966 over free RGFP966 thus reflects the ability of the nanocarrier to overcome the poor brain penetration and rapid clearance that limit free drug bioavailability. Concurrently, brain edema was markedly attenuated in the CD-TLNP@RGFP966 group (75.9%) relative to both the model Control and RGFP966 group (81.6%) (Figure 4(D)). This indicates that CD-TLNP@RGFP966 can effectively reduce BBB disruption and fluid extravasation in the ischemic region. Beyond direct tissue protection, CD-TLNP@RGFP966 conferred significant systemic benefits, including a milder reduction in body weight and a substantially higher survival rate (85.7% at 14 days post-tMCAO) compared to all other treatment groups (model Control: 42.9%; free RGFP966: 57.1%) (Figure 4(E,F)). The improved survival and weight maintenance likely stem from the combined effects of reduced infarct burden, attenuated cerebral edema, and diminished systemic inflammatory responses – the latter being particularly important as post-stroke systemic immunosuppression often predisposes animals to fatal infections. The blank carrier (CD-TLNP) showed no therapeutic effect, confirming that the observed benefits were drug-dependent and delivery-enhanced. Collectively, these results underscore that the synergistic combination of T7-mediated brain targeting and pH-triggered drug release is critical for maximizing the therapeutic potential of RGFP966 in IS, as it ensures efficient delivery to the ischemic lesion and controlled release, thereby amplifying its neuroprotective efficacy through targeted HDAC3 inhibition and downstream modulation of inflammatory and apoptotic pathways.

### 3.5. CD-TLNP@RGFP966 alleviated neuroinflammation via modulating microglial polarization

**Figure 5.**
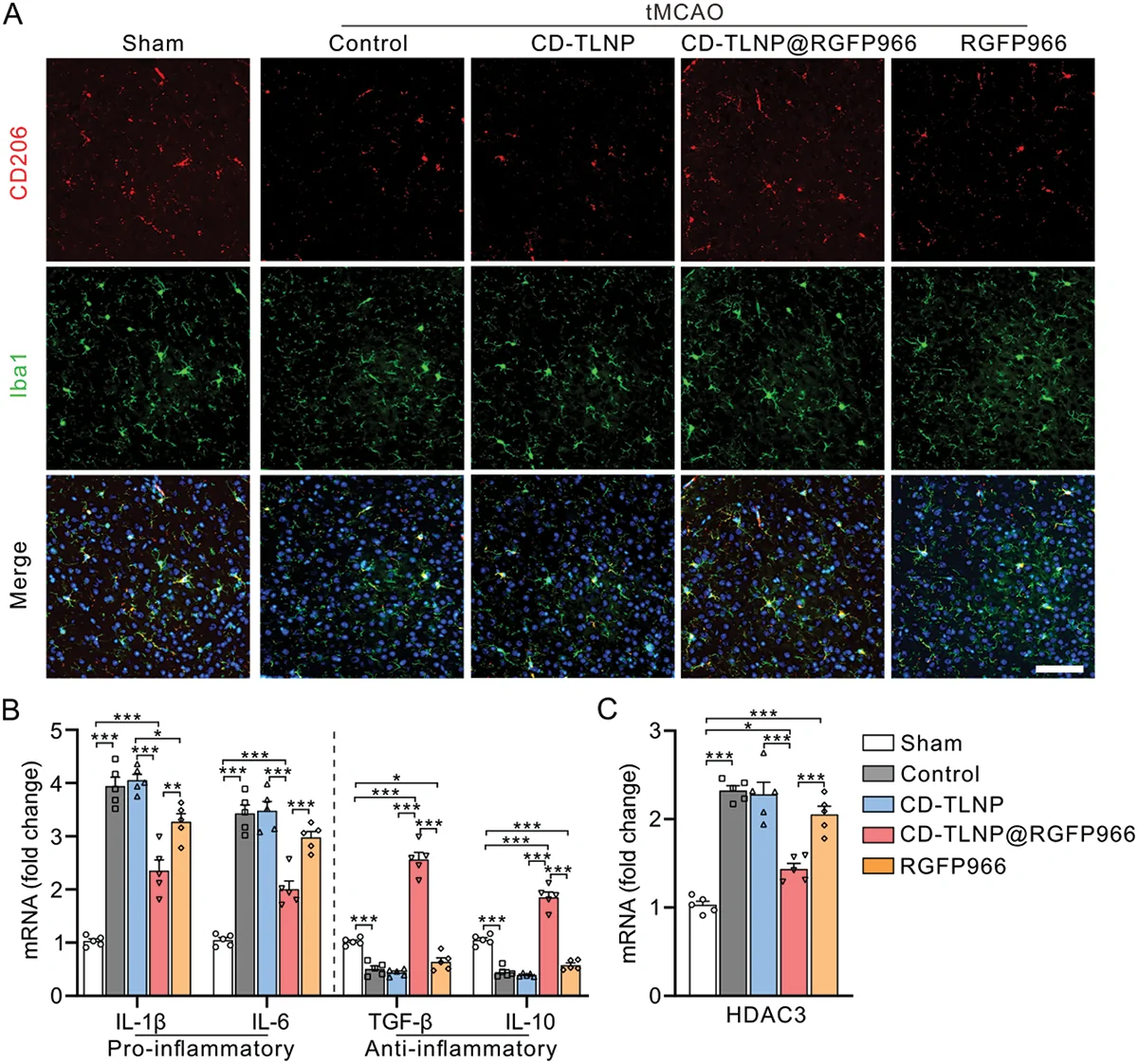
CD-TLNP@RGFP966 alleviates neuroinflammation by inhibiting HDAC3 in microglia. (A) Representative immunofluorescence images of M2-type (CD206) microglia in damaged brain tissues after different treatments. Scale bar: 50 µm. (B) Levels of pro-inflammatory factors (IL-1β, IL-6) and anti-inflammatory factors (TGF-β, IL-10) in damaged brain tissues by RT-qPCR. (C) Microglia from the injured brain region were isolated using magnetic bead-based sorting, followed by the detection of HDAC3 expression via RT-qPCR. *N* = 5. Data were expressed as mean ± SEM. *p < .05, **p < .01, ***p < .001.

To further explore the molecular mechanism underlying the neuroprotective effect of CD-TLNP@RGFP966, we evaluated its role in regulating microglial polarization and neuroinflammation in tMCAO mice. Immunofluorescence staining revealed distinct microglial polarization profiles. The results showed that the anti-inflammatory M2-type cells in the control group were scarce. In contrast, CD-TLNP@RGFP966 treatment significantly increased the number of CD206^+^ cells, confirming that it can effectively promote the transformation of microglia to the anti-inflammatory M2 phenotype, which is beneficial for clearing cell debris, inhibiting pro-inflammatory responses and promoting tissue repair (Figure 5(A)). The anti-inflammatory effect was corroborated at the cytokine level by RT-qPCR. CD-TLNP@RGFP966 treatment significantly downregulated the mRNA expression of pro-inflammatory factors (IL-1β, IL-6) and concurrently upregulated that of anti-inflammatory factors (TGF-β, IL-10) in the injured brain tissue, further verifying that the nanocarrier can effectively alleviate post-ischemic neuroinflammation (Figure 5(B)). This coordinated cytokine modulation is consistent with HDAC3 inhibition, as HDAC3 normally represses the transcription of anti-inflammatory genes while permitting NF-κB-driven pro-inflammatory gene expression [13–15]. By reversing this epigenetic balance, CD-TLNP@RGFP966 effectively alleviates post-ischemic neuroinflammation. Mechanistically, the expression of HDAC3 mRNA in sorted microglia was significantly lower in the CD-TLNP@RGFP966 group (relative expression, 1.4) than in the model Control (2.3), and this inhibitory effect was superior to that of free RGFP966 (2.1) (Figure 5(C)). Taken together, these results demonstrate that the delivery system efficiently targets and inhibits HDAC3 specifically in microglia, thereby reprogramming the inflammatory phenotype balance, attenuating post-ischemic neuroinflammation, and providing a key molecular mechanism for its observed neuroprotective effects.

### 3.6. CD-TLNP@RGFP966 promoted the recovery of neurological function

**Figure 6.**
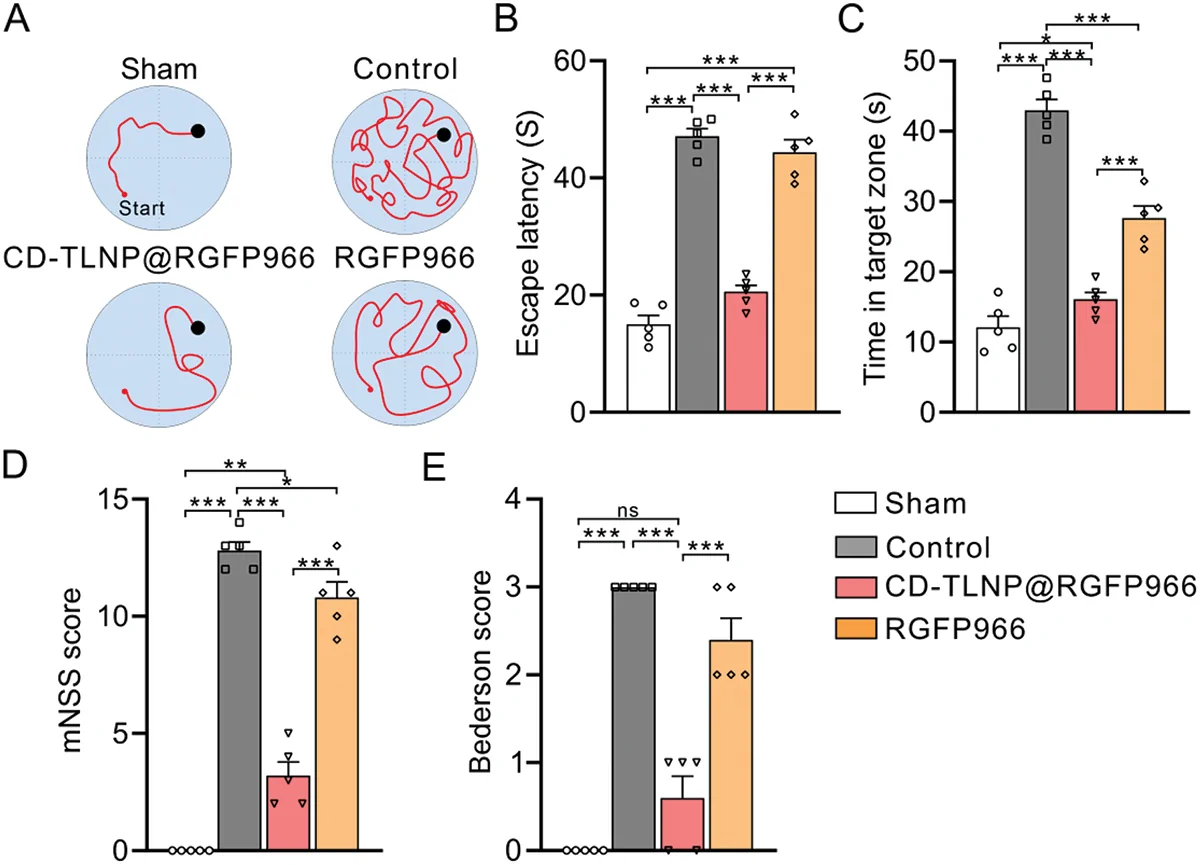
CD-TLNP@RGFP966 significantly promoted neurological functional recovery in tMCAO mice. (A) Representative swimming routes, (B) Escape latency and (C) Retention time in the target zone for different groups of mice at 7 days after stroke. (D) The mNSS score and (E) Bederson score for different groups of mice at 7 days after stroke. *N* = 5. Data were expressed as mean ± SEM. ns, no statistical significance, *p < .05, **p < .01, ***p < .001.

The therapeutic efficacy of CD-TLNP@RGFP966 was conclusively demonstrated by behavioral and functional analyses, which are critical indicators of the clinical relevance of stroke therapy. Cognitive assessment via the Morris water maze test, a classic method to evaluate spatial learning and memory ability in stroke models, revealed a strikingly shorter escape latency in the CD-TLNP@RGFP966 group (20.8 s) than in both the model control (47.2 s) and free RGFP966 (44.3 s) groups, accompanied by increased target quadrant dwell time and more direct swim paths (Figure 6(A–C)). These indicatedsubstantial restoration of spatial learning and memory function in the CD-TLNP@RGFP966-treated mice. Concomitantly, neurological deficits were robustly attenuated, as evidenced by significantly lower scores in both the mNSS and Bederson tests in the CD-TLNP@RGFP966 group compared to the model control, with its efficacy surpassing that of free RGFP966, confirming that the nanocarrier can effectively improve motor function, sensory function, and coordination ability in tMCAO mice (Figure 6(D,E)). The superior neurological recovery is likely underpinned by the reduced infarct volume and attenuated cerebral edema observed earlier, as smaller tissue damage directly translates to better preservation of neural circuitry and motor pathways. These behavioral and functional improvements collectively demonstrate the superior therapeutic effect of CD-TLNP@RGFP966 in mitigating post-stroke deficits, highlighting the synergistic enhancement achieved by its targeted delivery and pH-responsive release strategy, which ensures that RGFP966 exerts its anti-inflammatory and neuroprotective effects specifically within the ischemic brain region, ultimately promoting neurological function recovery.

## 4. Conculsion

In summary, this study successfully constructed a pH-responsive brain-targeted lipid nanoparticle delivery system CD-TLNP@RGFP966. The nanoparticle exhibited favorable physicochemical properties and biocompatibility. It can efficiently penetrate the BBB and accumulate at the lesion site. By inhibiting the expression of HDAC3 in microglia, it promotes the M2-type polarization of cells, thereby alleviating the neuroinflammation after IS and promoting the recovery of neural function. This work proposes a safe and effective nanotechnology strategy for targeted anti-inflammatory treatment in stroke. Nevertheless, this study has several limitations. The therapeutic time window was confined to the acute/subacute phase in a rodent model, leaving its long-term efficacy and safety unclear [34]. Moreover, the precise downstream signaling pathways of HDAC3 inhibition and the potential benefit of co-delivering other neuroprotective agents remain unexplored [35]. Future studies should therefore evaluate long-term outcomes [36], validate efficacy in more clinically relevant models (e.g. aged or comorbid animals), elucidate the complete HDAC3-regulated signaling network, and explore combination therapies. These steps are crucial for advancing this nanoplatform toward clinical translation.
